# Supplementary material for: The interactome of intact mitochondria by cross-linking mass spectrometry provides evidence for coexisting respiratory supercomplexes
Source: Mol Cell Proteomics. 2017 Dec 8;17(2):216–32. doi: 10.1074/mcp.RA117.000470 (PMC5795388; doi:10.1074/mcp.RA117.000470)
Supplement: Supplemental Data [file supp_RA117.000470_133922_0_supp_23978_fzffwf.zip › spectra_annotation/mito_DR_spectra_annotation/465-1-8-1-5-1.pdf]

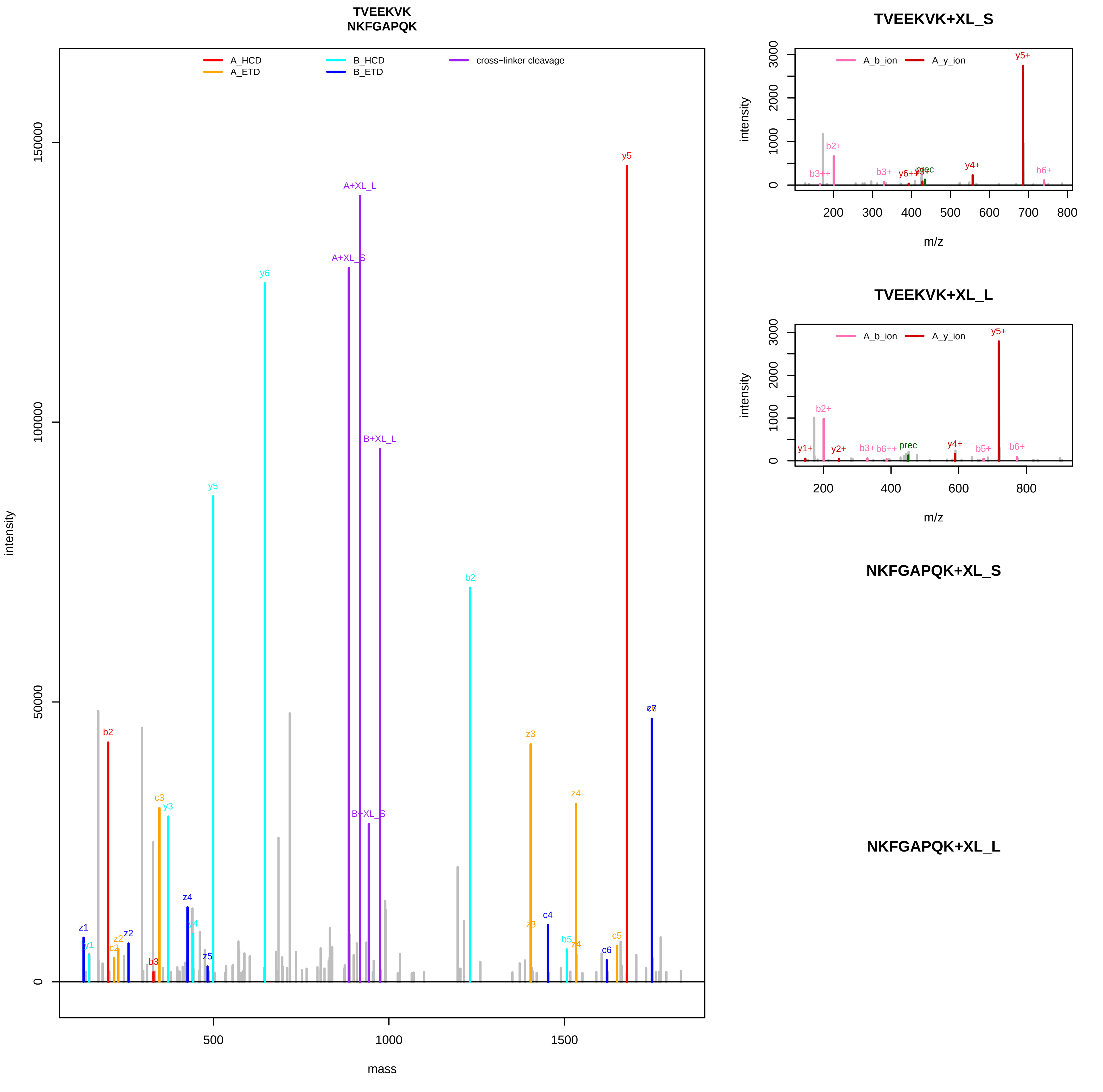
TVEEKVK
NKFGAPQK
A_HCD
A_ETD
B_HCD
B_ETD
cross−linker cleavage
intensity
mass
y5
A+XL_L
A+XL_S
y6
B+XL_L
y5
b2
b2
c7
z3
c3
y3
B+XL_S
z4
z3
c4
z1
y4
z2
z2
y1
c2
b3
z5
b5
z4
c6
c5
TVEEKVK+XL_S
A_b_ion
A_y_ion
y5+
b2+
y4+
b3++
b3+
y6++
prec
b6+
m/z
TVEEKVK+XL_L
A_b_ion
A_y_ion
y5+
b2+
y1+
y2+
b3+
b6++
prec
y4+
b5+
b6+
m/z
NKFGAPQK+XL_S
NKFGAPQK+XL_L
